# Usefulness of the MADIT-ICD Benefit Score in a Large Mixed Patient Cohort of Primary Prevention of Sudden Cardiac Death

**DOI:** 10.3390/jpm12081240

**Published:** 2022-07-28

**Authors:** Kevin Willy, Julia Köbe, Florian Reinke, Benjamin Rath, Christian Ellermann, Julian Wolfes, Felix K. Wegner, Patrick R. Leitz, Philipp S. Lange, Lars Eckardt, Gerrit Frommeyer

**Affiliations:** Clinic for Cardiology II: Electrophysiology, University Hospital Münster, 48149 Münster, Germany; julia.koebe@ukmuenster.de (J.K.); florian.reinke@ukmuenster.de (F.R.); benjamin.rath@ukmuenster.de (B.R.); christian.ellermann@ukmuenster.de (C.E.); julian.wolfes@ukmuenster.de (J.W.); felix.wegner@ukmuenster.de (F.K.W.); patrick.leitz@ukmuenster.de (P.R.L.); philipp-sebastian.lange@ukmuenster.de (P.S.L.); lars.eckardt@ukmuenster.de (L.E.); gerrit.frommeyer@ukmuenster.de (G.F.)

**Keywords:** ICD therapy, sudden cardiac death, risk

## Abstract

Background: Decision-making in primary prevention is not always trivial and many clinical scenarios are not reflected in current guidelines. To help evaluate a patient’s individual risk, a new score to predict the benefit of an implantable defibrillator (ICD) for primary prevention, the MADIT-ICD benefit score, has recently been proposed. The score tries to predict occurrence of ventricular arrhythmias and non-arrhythmic death based on data from four previous MADIT trials. We aimed at examining its usefulness in a large single-center register of S-ICD patients with various underlying cardiomyopathies. Methods and results: All S-ICD patients with a primary preventive indication for ICD implantation from our large single-center database were included in the analysis (*n* = 173). During a follow-up of 1227 ± 978 days, 27 patients developed sustained ventricular arrhythmias, while 6 patients died for non-arrhythmic reasons. There was a significant correlation for patients with ischemic cardiomyopathy (ICM) (*n* = 29, *p* = 0.04) to the occurrence of ventricular arrhythmia. However, the occurrence of ventricular arrhythmias could not sufficiently be predicted by the MADIT-ICD VT/VF score (*p* = 0.3) in patients with (*n* = 142, *p* = 0.19) as well as patients without structural heart disease (*n* = 31, *p* = 0.88) and patients with LV-EF < 35%. Of the risk factors included in the risk score calculation, only non-sustained ventricular tachycardias were significantly associated with sustained ventricular arrhythmias (*p* = 0.02). Of note, non-arrhythmic death could effectively be predicted by the proposed non-arrhythmic mortality score as part of the benefit score (*p* = 0.001, *r* = 0.3) also mainly driven by ICM patients. Age, diabetes mellitus, and a BMI < 23 kg/m^2^ were key predictors of non-arrhythmic death implemented in the score. Conclusion: The MADIT-ICD benefit score adds a new option to evaluate expected benefit of ICD implantation for primary prevention. In a large S-ICD cohort of primary prevention, the value of the score was limited to patients with ischemic cardiomyopathy. Future research should evaluate the performance of the score in different subgroups and compare it to other risk scores to assess its value for daily clinical practice.

## 1. Introduction

The subcutaneous ICD (S-ICD) (Boston Scientific, Natick, Massachusetts) is widely accepted as a valuable alternative to transvenous ICDs in a variety of clinical constellations requiring ICD therapy [1,2,3,4,5,6]. Quite recently, the highly anticipated results of the PRAETORIAN trial have been published, showing non-inferiority of the subcutaneous ICD compared to transvenous ICDs with regard to safety in a randomized controlled trial [7].

The MADIT-ICD benefit score is a new score that has recently been developed on the basis of the landmark MADIT trials to predict the individual patient risk for a sudden cardiac death and/or appropriate ICD therapy on the one hand and a non-sudden death on the other hand [8]. This may be of particular clinical importance as several recent studies have questioned the benefit of ICD therapy for primary prevention in the era of modern heart failure therapy [9,10,11]. One of the most recent trials is the randomized controlled DANISH trial, in which patients with a non-ischemic cardiomyopathy without an ICD had a non-inferior all-cause mortality in comparison to patients implanted with a ICD, although there was a significantly higher risk for sudden cardiac death in patients without an ICD [12].

The MADIT-ICD benefit score was developed on the basis of study populations from the MADIT trials of about 4500 ICD patients with a primary preventive indication for ICD implantation using a regression model identifying eight predictors of sudden death (male gender, age < 75 years, prior non-sustained VT, heart rate > 75 bpm, systolic blood pressure < 140 mmHg, ejection fraction < 25%, prior myocardial infarction and atrial arrhythmia) and seven predictors of non-sudden cardiac death in this cohort (age > 75 years, diabetes mellitus, body mass index < 23 kg/m^2^, ejection fraction < 25%, heart failure NYHA class II or worse, missing cardiac resynchronization therapy and atrial arrhythmia). These two scores directly point out the risk for SCD (occurrence of VT/VF during follow-up) on the one and non-arrhythmic death on the other hand. In addition, the two scores were combined to a third personalized ICD benefit score ranging from 0–100 with values from 76–100 indicating the highest benefit, 26–75 an intermediate benefit, and <26 lowest benefit of ICD implantation for primary prevention. The score showed good validity in this cohort and could show a high predictive value for life threatening events. As these scores are based on a proof-of-principle study in a typical MADIT collective with most patients suffering from ischemic cardiomyopathy, we sought to examine the usefulness of the score for a cohort of primary preventive ICD patients with different heart disease to further characterize its value in daily clinical practice.

## 2. Materials and Methods

The study was conducted in accordance with the guidelines of the Declaration of Helsinki. As it was a solely retrospective study, no additional vote from the local ethics committee was obtained. Patients were verbally informed about possibly being included in research projects anonymously when being treated in the University Hospital. No informed consent was obtained. Between June 2010 and January 2021, a total of 371 S-ICD systems were implanted at our institution. In the present single-center retrospective study, we enrolled all patients (*n* = 173, 46.6%) with primary preventive indication for S-ICD implantation. Indication for ICD implantation was in accordance to current ESC guidelines. Patient characteristics are summarized in Table 1. Prior to implantation, S-ICD screening was performed with the automated screening tool. Patients were considered eligible for S-ICD implantation if there was at least one suitable vector. All patients were scheduled for an intraoperative defibrillation test. In case of an unsuccessful test, the shock vector was changed to reversed polarity, the shock energy was raised, or, if necessary, system components were repositioned intraoperatively using fluoroscopy. We used a dual-zone ICD programming, setting the VT zone to 200–220 bpm and the VF zone to 240–250 bpm. For follow-up, patients were examined at six weeks after implantation and every three to six months subsequently. Adverse events were documented during regular follow-up in three- to six-month intervals.

Data needed to determine VT/VF-score and non-arrhythmic death score were obtained from medical charts and history. Share of missing for each variable was examined. Datasets were complete for all variables analyzed in all patients. For calculation of the individualized risk score, the automated risk calculator provided online under https://is.gd/madit (accessed on the 18th March 2021) on the website of the University of Rochester was used as described in the original manuscript [8].

Data transformation and statistical analysis was performed using GraphPad PRISM 6.0 (San Diego, CA, USA) and the SPSS Statistics, version 20.0 (SPSS, Inc., Chicago, IL, USA). Continuous variables are presented as mean and standard deviation (SD), while categorical data are expressed as frequencies. For prediction of the occurrence of VT/VF or non-sudden death, multiple linear regression analyses were performed. A *p*-value of <0.05 was considered statistically significant.

## 3. Results

In total, we included 173 patients with S-ICD implanted for primary prevention. 124 patients were male (71.7%) with a mean age of 43.2 ± 16.0 years. The mean follow-up duration was about three and a half years (1227 ± 978 days (see Table 1). Dilated cardiomyopathy (24.3%), hypertrophic cardiomyopathy (21.4%), ischemic cardiomyopathy (17.3%), and electrical heart disease (15.6%) were the major indications for S-ICD implantation. Mean left ventricular ejection fraction was 44.9 ± 16.1%.

In total, 27 (15.6%) patients received an appropriate ICD therapy during follow-up, while 6 patients (3.5%) died during follow-up due to non-sudden causes, mostly due to heart failure or sepsis.

The mean VT/VF-score was 7.2 ± 1.8 ranging from 4–12 points, while mean score for non-arrhythmic mortality was 1.9 ± 1.8 ranging from 0–8 points. These scores resulted in a mean personalized benefit score of 59.5 ± 17.6 ranging from 20–87. In total, 59 patients were sorted to the high benefit group, 101 patients to the intermediate benefit group, and 13 patients to the low benefit group, respectively.

Statistical analysis revealed no significant correlation between neither the benefit score (*p* = 0.62) nor the VT/VF score (*p* = 0.3) with the occurrence of appropriate therapies during follow-up. This finding was also confirmed when ordering the patients according to their respective subgroups (high/intermediate/low benefit) since there was no significant correlation in any of these groups. In absolute numbers, there were 8 shocks in 59 patients from the highest group (13.6%), 18 shocks in 101 patients from the intermediate group (17.8%), and 1 shock in 13 patients from the low-risk group (7.7%). Differences did not reach statistical significance. Please see Table 2 for a detailed description.

### 3.1. Subanalysis for Patients with and without Structural Heart Disease

Furthermore, as the MADIT cohorts were used for validation and indeed consist of patients with structural heart diseases (SHD), we divided our cohort in patients with (*n* = 142) and without SHD (*n* = 31). For these subgroups statistical analysis revealed no predictive value of the VT/VF-Score (SHD *p* = 0.19; no SHD *p* = 0.88) nor the benefit score (SHD *p* = 0.69; no SHD *p* = 0.74) (for detailed information please see Table 3).

### 3.2. Subanalysis for Patients with Ischemic Cardiomyopathy

When further dividing between patients with ischemic (ICM) and non-ischemic cardiomyopathy (NICM), however, there was a significant predictive value of the VT/VF-Score for appropriate ICD interventions (*p* = 0.04) but not for the benefit score (*p* = 0.45) in ICM patients. For NICM patients, there was no significant predictive value for both scores (VT/VF-score: *p* = 0.55, benefit score: *p* = 0.86).

While the benefit score did not significantly predict non-arrhythmic death as well (*p* = 0.25), the non-arrhythmic death score indeed did (*p* = 0.0001, *r* = 0.3).

These results were also driven by ICM patients (*p* < 0.001), while there was no significant correlation in NICM patients (*p* = 0.18). In the group of patients, no patient died during follow-up, so that no statistical analysis regarding this outcome could be performed.

To identify the predictive value of the factors implemented in the score, we performed a logistic regression analysis with either appropriate therapy or non-arrhythmic death as dependent variable.

Concerning the prediction of appropriate therapy, only the presence of prior non-sustained VT had a significant impact (*p* = 0.02); none of the other factors reached true or borderline statistical significance (for detailed information please see Table 2).

Taking a look at the different risk groups, there were only two factors in the intermediate risk group significantly associated with appropriate therapy: NYHA II or worse and high heart rate, which did not play a role in the main analysis (NYHA II: *p* = 0.5, heart rate > 75 bpm: *p* = 0.72 in the main analysis, respectively).

For the prediction of non-arrhythmic mortality, the analysis revealed three factors significantly associated with the occurrence of non-arrhythmic death. These factors were an age < 75 years, which had a strong negative correlation with non-arrhythmic death (*p* = 0.0001) and the presence of diabetes mellitus (*p* = 0.01) and a BMI < 23 kg/m^2^ (*p* = 0.005). The relevance of these factors could be confirmed in every one of the three subgroups.

### 3.3. Subanalysis for Patients with LV-EF < 35% (MADIT Criterion)

As the MADIT-ICD benefit score was derived from data of patients included in the MADIT trials, we also regrouped patients according to the fulfillment of the MADIT criteria for a primary preventive ICD implantation, namely a persisting LV-EF < 35%.

For the prediction of VT/VF in patients with a LV-EF < 35%, regression analysis revealed no significant predictor for the occurrence of VT/VF during follow-up, while high age and low BMI could significantly predict non-arrhythmic death (*p* < 0.001 for age and *p* = 0.008 for BMI respectively). For patients with LVEF > 35%, there was also no parameter associated with VT/VF during follow-up. As there were no non-arrhythmic deaths in this group during follow-up, parameters were not analyzed for this endpoint. 

## 4. Discussion

In our large S-ICD patient cohort with an ICD implanted for primary prevention, the MADIT benefit score did work for patients with ICM, while it could not facilitate prediction of adequate ICD therapies in other constellation. This was also true for patients with a persisting LV-EF < 35% not fulfilling MADIT inclusion criteria despite optimal medical therapy.

These findings may be explained by several reasons. First and probably most importantly, our S-ICD collective differs significantly from the patient population in which the benefit score was developed and validated. Our study cohort was younger and included more patients with hypertrophic cardiomyopathy and channelopathies apart from patients with ischemic cardiomyopathy [8,13,14,15,16]. For instance, a low blood pressure <140/90 mmHg is a key risk factor for the occurrence of VT/VF in the benefit score. However, in our patient collective, only 16 of 173 patients had a blood pressure below this threshold. All other patients received two points on the VT/VF score, so that it has to be assumed that in younger patient cohorts, including more patients without ischemic cardiomyopathy and classical risk factors such as arterial hypertension, the arrhythmogenic risk is considerably overestimated. However, in subgroup analysis, we were able to show that the VT/VF-Score and the MADIT ICD benefit score did not predict ventricular arrhythmias in patients with other structural heart diseases not covered by the MADIT inclusion criteria.

Furthermore, other patient characteristics and risk factors such as chronic renal failure as a strong predictor of non-arrhythmic death [11,17], a positive family history for sudden cardiac death which could be especially important in young patients with genetically determined cardiomyopathy [18,19], and malignancies as mortality-influencing factors being a major risk factor for non-arrhythmic death have not been considered yet in the risk score. For instance, in two recently published studies examining the genetic background in adult patients with dilated cardiomyopathy, the presence and type of underlying gene mutation had a substantial role in clinical outcome [20,21]. Especially in patients with dilated cardiomyopathy, cardiac MRI and distribution and extent of late gadolinium enhancement have also been shown to be very useful tools for risk stratification [22]. In contrast, e.g., a BMI < 23 kg/m^2^ does not seem not to be a suitable parameter for evaluating the risk of VT/VF in a young woman with long QT syndrome, while it is certainly helpful in an older patient cohort indicating cachexia and multimorbidity [20]. Surprisingly, despite the young mean age, the BMI was also a significant predictor of non-arrhythmic death in our cohort, underlining the potential role of this parameter.

The only parameter used in the VT/VF score consistently being associated with VT/VF in all risk groups was non-sustained VT (nsVT), which is in line with previous studies [3,23,24]. Therefore, nsVT as a precursor of later sustained VT may justify more intensified monitoring or deciding in favor of ICD implantation even in patients with LV-EF >35% if the patients have e.g., additional risk factors such as extensive late gadolinium enhancement in cardiac MRI or a positive family history for sudden cardiac death. Furthermore, documented nsVT can also lead to the consideration of EP study with programmed ventricular stimulation for further risk stratification, as inducibility of VT or VF have been shown to independently predict later ICD therapies [25].

Regarding age, there is evidence that although cardiovascular morbidity and mortality is increasing over time, patients often do not benefit from ICD implantation for primary prevention. Bilchick et al. demonstrated in a very large patient cohort of about 100,000 patients with heart failure that about one quarter of patients having been implanted with an ICD did not have any survival benefit from ICD implantation, with older age being one of the main predictors of not profiting [26,27]. This result is probably explained by other comorbidities competing with arrhythmic mortality in older patients [28]. This relationship of sudden to non-sudden death decreasing with age was already published by Kahn et al. in 2004 [29].

Furthermore, there are possibly additional risk factors not represented in the benefit score. For instance, advanced renal disease without or with chronic hemodialysis in a patient collective is associated with high morbidity and mortality. Evidence for a missing reduction of mortality by ICD implantation for primary prevention in this patient cohort has been initially observed in small observational cohorts [30] and strengthened by the ICD2-trial, a randomized controlled trial, in 2017 [11]. Therefore, it might be worth looking at a possible influence of renal function on benefit score in future research as advanced renal failure could be a relevant risk factor for non-sudden cardiac death [31] An interesting approach has been presented by Gatzoulis et al. in the PRESERVE EF study, using a two-step-approach combining initial ECG screening leading to programmed ventricular stimulation in case of ECG abnormalities in post-MI-patients with an LV-EF > 40% [32]. In case of inducibility, patients were offered an ICD. The authors could show convincing results, especially with a very high negative predictive value for a negative EP study during follow-up. Other authors have also highlighted the potential benefit for two-step approaches in non-ischemic patient cohorts [33,34]. This might be a hint for the value of stepwise and more elaborated approaches for other patient cohorts as well.

## 5. Limitations

This study has limitations caused by its retrospective nature. Furthermore, it has to be underlined that follow-up was not equally long for all patients. Besides, the MADIT-ICD benefit score is a novel tool which has not yet been evaluated in other populations, such that this study is a first step to testing it in a younger patient cohort with a large variety of underlying cardiomyopathies rather than a classical ischemic cardiomyopathy patient cohort. Furthermore, the patient cohort was rather small so that the limited usefulness of the score in this patient cohort could also be explained by the missing statistical power. In addition to that, patients with an S-ICD are, despite the growing rates of implantation in elderly patients and patients with ICM, a selected patient cohort. This must be taken into consideration when evaluating the results of our study. Another interesting aspect for consideration would have been the structured record of cardiac biomarkers such as natriuretic peptides or Troponin levels. As there was no structured recording, we decided not to add these biomarkers to the analysis but think that it could be of interest in future studies. Moreover, studies with larger mixed patient cohorts that include patients with transvenous ICDs have to be performed to achieve a higher generalizability of the results.

## 6. Conclusions

The recently developed MADIT-ICD benefit score is a new tool for benefit evaluation of primary prevention ICD implantation. One of its major advantages is the opportunity to calculate it with a small amount of easily available information. Of note, in our population, its diagnostic yield was limited to patients with ischemic cardiomyopathy. As many of the parameters influencing the score result (blood pressure, heart rate, BMI, age < 75 years) were very different from the validation cohorts, it might therefore be necessary to further advance the score by examining additional different patient cohorts or adopt the score with different parameters for different populations. The option of having another tool to evaluate the risk–benefit relation is indeed very attractive, so further development of risk scores is desirable.

## Figures and Tables

**Table 1:** Baseline characteristics of the patient cohort.

Baseline Characteristics	Total (*n* = 173)
Male (*n*)	124 (71.7%)
Age (years)	43.2 ± 16.0
Left ventricular ejection fraction (%)	44.9 ± 16.1
Follow-up duration (days)	1227 ± 9782
Underlying heart disease	
ICM	30 (17.3%)
DCM	42 (24.3%)
Electrical heart disease	27 (15.6%)
HCM	37 (21.4%)
Congenital heart disease	14 (8.1%)
Other	23 (13.3%)

**Table 2:** MADIT-ICD benefit score parameters as predictors for the occurrence of VT/VF in the whole cohort.

Parameter	*p*-Value	*p*-Value
	(Occurrence of VT/VF)	(Non-Arrhythmic Mortality)
Age (years)	0.281	0.019 *
Age < 75 years	0.255	0.001 *
Gender	0.635	0.271
LV-EF	0.737	0.205
Atrial Arrhythmia	0.681	0.677
Heart rate > 75 bpm	0.522	0.723
Systolic blood pressure < 140 mmHg	0.754	0.997
Prior myocardial infarction	0.710	0.518
Prior non-sustained VT	0.021 *	0.573
NYHA ≥ 2	0.498	0.489
Diabetes mellitus	0.905	0.010 *
BMI < 23 kg/m^2^	0.210	0.005 *

Analysis of MADIT-ICD benefit score parameters for the prediction of the occurrence of VT/VF as well as non-arrhythmic death in the cohort. A p-value <0.05 is deemed statistically significant and indicated with an asterixis (*).

**Table 3:** Comparison of the patients with and without structural heart diseases (SHD) within the trial.

Parameter	SHD (*n* = 142)	No SHD (*n* = 31)
Age (years)	44.6 ± 15.9	36.4 ± 14.6
Male gender (%)	63.6%	45.2%
LV-EF (%)	41.7 ± 15.8	59.6 ± 6.3
Appropriate ICD therapy (*n*/%)	22 (15.5%)	5 (16.1%)
*p*-value for correlation of ICD therapy	0.19	0.88
with VT/VF score		
Ischemic Cardiomyopathy	0.04	
Non-ischemic Cardiomyopathy	0.55	
*p*-value for correlation of ICD therapy	0.69	0.74
with ICD benefit score		
Ischemic Cardiomyopathy	0.45	
Non-ischemic Cardiomyopathy	0.86	
Non-arrhythmic death (*n*/%)	6 (4.2%)	0
*p*-value for correlation of non-arrhythmic	0.001	n.a.
Death with non-arrhythmic mortality		
Risk score		
Ischemic Cardiomyopathy	0.001	
Non-ischemic Cardiomyopathy	0.18	

## Data Availability

Original and raw data can be obtained upon reasonable request from the corresponding author under kevin.willy@ukmuenster.de.
